# Alzheimer’s Disease and Its Potential Alternative Therapeutics

**DOI:** 10.4172/2161-0460.1000477

**Published:** 2019-09-13

**Authors:** Brent Kisby, Juliet T Jarrell, M Enes Agar, David S Cohen, Eric R Rosin, Catherine M Cahill, Jack T Rogers, Xudong Huang

**Affiliations:** Neurochemistry Laboratory, Department of Psychiatry, Massachusetts General Hospital and Harvard Medical School, Charlestown, MA, USA

**Keywords:** Alzheimer’s disease, Neurogenesis, Amyloid precursor protein, Amyloid-beta, Tau protein, Alternative AD therapeutics

## Abstract

Alzheimer’s Disease (AD) is a chronic neurodegenerative disease that affects over 5 million individuals in the United States alone. Currently, there are only two kinds of pharmacological interventions available for symptomatic relief of AD; Acetyl Cholinesterase Inhibitors (AChEI) and N-methyl-D-aspartic Acid (NMDA) receptor antagonists and these drugs do not slow down or stop the progression of the disease. Several molecular targets have been implicated in the pathophysiology of AD, such as the tau (τ) protein, Amyloid-beta (Aβ), the Amyloid Precursor Protein (APP) and more and several responses have also been observed in the advancement of the disease, such as reduced neurogenesis, neuroinflammation, oxidative stress and iron overload. In this review, we discuss general features of AD and several small molecules across different experimental AD drug classes that have been studied for their effects in the context of the molecular targets and responses associated with the AD progression. These drugs include: Paroxetine, Desferrioxamine (DFO), N-acetylcysteine (NAC), Posiphen/-(−)Phenserine, JTR-009, Carvedilol, LY450139, Intravenous immunoglobulin G 10%, Indomethacin and Lithium Carbonate (Li_2_CO_3_).

## Introduction

Alzheimer’s Disease (AD) is a chronic neurodegenerative disease that typically occurs in those aged 65 years and older [1]. It presents with a broadly related pathophysiology as Frontotemporal Dementia (FTD) and Amyotrophic Lateral Sclerosis (ALS), also known as Lou Gehrig’s disease. AD is a senile dementia with its pathology occurring up to 18 years before its definitive diagnosis [2]. In 2017, the Alzheimer’s association published a 2017 Facts and Figures report indicating that approximately 5.5 million people in America have Alzheimer’s and of those 5.5 million, 5.3 million are 65 years of age or older. Additionally, by 2050, the number of people that have Alzheimer’s may triple. The report also shows that, between 2000 and 2014, the death rate of Alzheimer’s has increased by 89%, by contrast, other public health conditions such as stroke and HIV have observed a 14% and 54% death rate decline, respectively.

AD and other types of dementia are characterized by a loss of ability to solve problems or maintain emotional control. Individuals with dementia experience personality changes and behavioral problems such as agitation and impaired intellectual functioning that interfere with normal activities and relationships. Dementia also generates hallucinations and delusions.

There are currently only a few FDA-approved treatments for AD, which belong to two classes of drugs: Acetyl cholinesterase Inhibitors (AChEI) and N-methyl-D-aspartic Acid (NMDA) antagonists. These treatments have been used for treating the symptoms of cognitive decline and language deficits and do not slow down or stop the progression of the disease. A drug that cures or at least slows down the disease is necessary, as Alzheimer’s is becoming pandemic.

Here we review select aspects of the AD pathogenesis, focusing on iron homeostasis and oxidative stress, dysregulation of the APP translation and processing, Amyloid-beta (Aβ) deposition, the proinflammatory cytokine Interleukin-1 beta (IL-1β), tau protein hyperphosphorylation and reduced neurogenesis. Moreover, we address a few compounds that provide alternatives to the current FDA approved drugs and target many different components and pathways of AD.

## General Features of AD

### Risk factors attributed to AD

There are many environmental and biological risk factors attributed to the pathogenesis of AD, which include, but are not limited to: chronic alcoholism, age, neuroinflammation, Apolipoprotein E fibrillation, iron dysregulation, cerebral ischemia, stress, lack of sleep, genetic mutations, mitochondrial dysfunction, iron overload and oxidative stress and metal dyshomeostasis (i.e., copper, aluminum, iron, manganese)[3–18].

### Pathogenesis of AD

AD is a highly complex disease with numerous features. A common generalization is that AD results from insoluble Aβ plaque formation in hippocampal neurons and neuro-fibrillations of tau protein created *via* tau hyperphosphorylation [19–21]. Eventually, these plaques and neuro-fibrillations cause neuronal apoptosis and neurodegeneration [20,21]. Although Aβ plaques and tau neuro-fibrillations are critically important features of AD, there are many other components of the disease as well, some still unknown that should be considered equally in the search for a cure.

#### Iron and oxidative stress:

Iron (Fe) is one of the redox-active transition metals and Fe, along with other metals, has been shown to promote the formation of Aβ plaques and engender neuronal oxidative stress [18]. The ability of Fe to induce oxidative stress is attributed to the valence state of iron (Fe) being reduced from Fe (III) to Fe(II) and this reduction is coupled with hydroxyl radical formations in the brain through the Fenton reaction [15,18]. As shown in multiple studies, the radical formations reduce the proliferation of Neural Stem Cells (NSCs) and neurogenesis in an AD brain [22–24]. Furthermore, oxidative stress has been known to cause tau neurofibrils, neurogenesis deterioration and increased ferritin levels that have been correlated with cognitive decline [25–28].

#### Amyloid precursor protein:

The Amyloid Precursor Protein (APP), which can generate Amyloid-beta (Aβ) through proteolysis, plays a vital role in synaptic formation, iron regulation, neural plasticity and neurogenesis [9,29–33]. The 5’ UTR region of the APP plays a role in APP expression and the formation of Aβ and it remains a possibility that these processes are accelerated in the presence of iron through a 5’-Untranslated Region (UTR) iron response element (IRE) in the APP transcript [34,35]. The 5’ UTR specific IRE RNA stem loop was first reported in 2002 and has since proven to present a target for chelators and other drugs that inhibit APP translation, such as desferrioxamine, clioquinol, VK-28, piperazine-1, phenserine, tetrathiomolybdate, dimercaptopropanol, paroxetine, azithromycin and a high throughput benzimidazole 5’UTR translation blocker designated as JTR-009 [35–39]. JTR-004, JTR-009, JTR-0013 were among the most potent compounds tested in the high throughput study that inhibit the 5’ UTR APP translation, with JTR-009 being the most potent blocker, whereas other endogenous compounds or hormones and amyloid expression such as glucocorticoids have been implicated in increasing APP translation [40].

#### β-amyloid plaques:

Beta-amyloid plaques are one of the two most distinguishing features of AD. There are two types of Aβ subtypes which have been implicated in causing AD progression, these mutations are Aβ_1/40_ and Aβ_1/42_. In the context of AD, Aβ has been known to cause insoluble plaques and inhibit neurogenesis by suppressing proliferation of NSCs, this suppression eventually leads to neuronal apoptosis *in vivo* [41–43]. The build-up of these plaques can create inflammation and oxidative stress [44,45]. A vast amount of research regarding the role of Aβ in Alzheimer’s already exists and this research is ongoing.

#### Tau and tauopathy:

The second distinguishing feature of AD other than beta-amyloid plaques is the appearance of tau neurofibrillary tangles. Tau is highly soluble microtubules associated protein that is part of a superclass of Microtubule Associated Proteins (MAP) which regulates neuronal microtubule within axons and are localized in dendrites in AD neuropathology [46]. AD is classified as a tauopathy, tauopathies are a group of neurodegenerative diseases that involve tau tangles. Some other tauopathies include ALS, FTD and Pick’s Disease [47–49].

Research about tau is ongoing; a recent report shows that tau protein causes a decline in neurogenesis. In this 12 month study, as tau levels increased, the level of neurogenesis in the hippocampus and Subventricular Zone (SVZ) decreased [50]. Furthermore, prion proteins (PrP^C^), which prevent cells from oxidative stress, interact with tau, but the mechanism and effects of these proteins are unclear, some evidence shows that these proteins stabilize tau and Aβ production, while other evidence suggests that the proteins can arrest APP translation and tau production [51,52]. Tau is regulated by 2 factors: Glycogen Synthase Kinase-3 (GSK-3β) and Cyclin-Dependent Kinase 5 (CDK5). GSK-3β and CDK5 regulate the activation of tau phosphorylation and this phosphorylation leads to tauopathy [53–55]. Researchers studying the inverse effects of GSK-3β have determined that GSK-3β inhibitors, such as Lithium Carbonate (Li_2_CO_3_), can inhibit the tauopathy in AD [56].

#### Interleukin-1 beta (IL-1β):

Proinflammatory cytokines have been implicated in AD pathogenesis. The most common of the cytokines with respect to AD pathology is Interleukin 1 beta (IL-1β). IL-1β is in the supergroup of Interleukin-1 (IL-1), which includes a plethora of other proinflammatory and anti-inflammatory cytokines. IL-1β is regulated by several factors including, but not limited to, caspase-1, IRAK1/2, transcriptional and translational level regulation and procaspase-1. Limited data indicates IL-1β’s role in iron regulation, but one study showed an increase in ferritin protein expression levels due to increased levels of IL-1β in the mitochondria [57]. IL-1β also has a connection to oxidative stress, it is spliced by caspase-1, which is downstream of the cellular oxidative stress pathway [58]. Further review of IL-1β’s biochemical properties can be found in Dinarello CA 2011[59].

IL-1β in the brain is localized within microglial cells, astrocytes and B and T cells within the periphery [60–63]. IL-1β has toxic effects because it serially increases APP translation and increases tau phosphorylation [64,65]. Aβ has also been classified as a proinflammatory peptide because it enhances production of pro IL-1β into mature IL-1β and this mature form can create microglial toxicity [66]. Recently, one study showed that a drug called Edaravone can alleviate this microglial toxicity and stop the release of proinflammatory cytokines such as IL-1β [67].

IL-1β can even trigger a positive, inflammatory feedback loop in some vertebrae cells [68]. It is not known whether increased IL-1β and microglial toxicity as a result of this positive feedback loop might precede Aβ production and plaque formation over the time span of AD pathogenesis and more research should be conducted about the relationship between IL-1β and AD, as IL-1β could be an AD drug target, IL-1β is already a drug target for autoimmune disorders [69].

Moreover, a groundbreaking study by Monje et al. showed that inhibiting inflammatory cytokines increases neurogenesis in hippocampal neurons and a more recent review suggests that IL-1β negatively affects neurogenesis, this research further implicates IL-1β’s role in neurogenesis and, ultimately, AD [70,71].

## Pathology of AD Effects on Neurogenesis

Neurogenesis in humans is found in the the Subventricular Zone (SVZ), the Dentate Gyrus (DG) of the hippocampus and the Olfactory Bulb (OLFB) [72,73]. Neurogenesis is the process of producing adult neurons from Neural Stem Cells (NSCs) [38]. Many studies suggest that neurogenesis is decreased in AD [73–76]. There are several biological factors that inhibit neurogenesis, such as IL-1β, serotonin depletion, mutations in presenilin-1 and normal aging and shortening of telomeres [19,43,77,78]. It has been hypothesized that since neurogenesis in humans is in the OLFB, early signs of AD progression can manifest in neurogenesis decline, where decreased smell correlates with AD advancement, thus, smell could be helpful in early detection of AD [79]. Moreover, tau presence in the OLFB also correlates with early stage AD [80]. Thus, further understanding of the molecular pathology and the decline of neurogenesis should advance our knowledge about the early signs of clinical AD.

## Current Small Molecule Treatments for AD

There are currently two classes of drugs that are used to treat the symptoms of AD: Acetylcholinesterase Inhibitors (AChEI) and N-Methyl-D-Aspartate (NMDA) receptor antagonists [81]. Acetylcholinesterase (AChE) is an enzyme which degrades acetylcholine (ACh) in the synapse [82]. The drugs that belong to the AChEI class are donepezil, rivastigmine and galantamine.

Decreased ACh in the AD brain has been well documented and evidence suggests that an AD-associated lowering of ACh activity in the basal forebrain leads to compromise of executive functions, ACh plays a role in certain cognitive abilities such as attention [83–86]. Studies also show a decrease in AChE activity in AD patients and that AChEI’s can reduce IL-1β and increase interleukin-4 (IL-4) [87,88]. There are several reviews on how AChE inhibitors work by arresting the activity of AChE, thus leading to increases in Ach [89–91].

By comparison, NMDA receptor antagonists are used to treat the toxic increases in Glutamate (Glu) within the brain [92]. Glu excitotoxicity has been hypothesized to play a pivotal role in AD pathology because excess Glu in synapses leads to neuronal apoptosis [93]. Memantine, at the time of this review, is the only NMDA antagonist available on the market. Memantine has been shown to be neuroprotective as well as increase LTP in CA1 hippocampal neurons [94–96]. Both NMDA antagonists and AChEI’s have been shown to have neurotrophic effects [97,98].

### Drawbacks to current AD treatments

Both AChEI’s and the NMDA antagonist memantine have a few benefits for AD patients, such as displaying mild neuroprotective properties inhibiting cognitive decline for severe AD patients and facilitating neurogenesis [87,99–103]. However, these drugs do not slow down or stop the progression of the disease, they simply try to relieve symptoms and they become less effective over time [86,104]. Because the current drugs are not curative, many new drugs are in development. According to a September 2017 report, at that time there were 150 drugs in the AD treatment development pipeline, in various clinical stages [105].

## Alternative Treatments for AD

There are many benefits and drawbacks to the following alternative treatments. These therapies are possible alternatives to AChEI’s and the NMDA antagonist memantine and have multiple implications for efficacy and improvement of cognition in AD patients. The main goal of these alternative therapeutic compounds is to manage the symptoms of AD and improve a patient’s quality of life, while also improving the underlying AD pathology *via* their molecular and biochemical properties. The issue is that definitive conclusions cannot be made about these drugs due to lack of research and/or conflicting research. However, the results from the research that has been done should shed light on more individualized therapies that can assist in AD pathology reversal and enhance hippocampal neurogenesis and memory.

We present several different novel treatment options entering the pipeline that have somewhat been or will be considered for AD or that have not been extensively studied, but have positive effects on different AD targets discussed in the first section of this review.

### Paroxetine and various selective serotonin reuptake inhibitors

Paroxetine (Paxil) is a Selective Serotonin Reuptake Inhibitor (SSRI) and is FDA approved for treating depression, mood disorders and anxiety disorders [106,107]. The drug works by inhibiting the reuptake of serotonin (5-HT) on the presynaptic neuron, which then increases 5-HT within the synapse. Paroxetine has been shown to enhance iron homeostasis and act as an inhibitor of the 5’-UTR for APP to generate anti amyloid efficacy [108]. Tucker et al. employed western blotting experiments to demonstrate a consistent 20% reduction in the steady-state levels of APP holoprotein in the brain cortex of paroxetine treated mice [109]. Likewise, according to Payton et al. paroxetine decreased intracellular APP translation [110].

Recent studies have looked at the implications of SSRI’s for enhancing cognitive abilities in the context of AD. SSRI’s have been shown to be beneficial to hippocampal function with chronic use because they increase NSCs both *in vivo* and *in vitro* through up-regulating phosphorylation of GSK-3β, which in turn modulates the 5-HT_1A_ receptor [111–113]. Fluoxetine and imipramine, two other SSRI’s, can reverse the loss of NSCs, so Paroxetine may have this capability as well [114]. Furthermore, Paroxetine has been shown to increase neurogenesis in human stem cells and in rats and improve memory after cerebral ischemia in a rat model [115–118]. Along with Paroxetine, other anti-depressants like amitriptyline, which is a tricyclic antidepressant, have been shown to increase levels of a non-toxic Aβ peptide and neurogenesis [119]. However, one study showed that Paroxetine does not increase total granular neurons or spatial memory [120]. More research needs to be done to determine the effects of Paroxetine and other antidepressant drugs on neurogenesis in AD patients specifically. A 2017 study has shown that Paroxetine can increase ROS, mitochondrial dysfunction and astrocyte apoptosis, so more research about the downsides of Paroxetine is also required [121].

### Desferrioxamine (DFO)

Desferrioxamine (DFO) is an FDA approved drug for treatment of iron overload from blood transfusions and it is a potent iron and aluminum chelator. DFO works by binding to metals to create iron displacement and this displacement ultimately prevents the Fenton reaction [122]. DFO has had several benefits for AD patients. First, daily doses of intramuscular (IM) DFO slowed the progression of AD by decreasing tau and either reduced inflammation or free radical production [49,57]. Second, DFO inhibited the tau protein from hyperphosphorylation *via* GSK-3β [123]. Third, the drug slowed cognitive decline in transgenic APP/PS1 mice and nontransgenic mice and increased memory in an AD rat model [124–126]. Finally, DFO has been proven to inhibit Aβ deposits and apoptosis in the brain of APP/PS1 double transgenic mice [127]. Iron chelators such as DFO are attractive agents that merit further investigation for use in AD. A recent study has shown that iron-chelating nanogels containing DFO components may be more effective for treating iron overload than DFO itself and more research about these nanogels is required [128,129].

### N-acetylcysteine (NAC)

NAC is currently an FDA approved antioxidant and it has been shown to suppress the inflammatory nuclear factor-kappa B (NF-kB), which has been implicated in AD [130,131]. NAC works by reducing reactive oxygen species and apoptosis and by modulating glutathione levels [132–134]. Another benefit of NAC is that it enables neuritogenesis and differentiation of stem cells [135]. One recent study showed that NAC reverses oxidative stress due to anxiety in a zebrafish model [136]. There are very few studies on the efficacy of NAC for treating AD and one concern about NAC is its low membrane permeability, but more information on the subject is needed [137].

### Posiphen/phenserine/JTR selective APP inhibitors

Phenserine both inhibits the 5’UTR of APP and also has AChEI properties, furthermore, it reduces APP expression in response to IL-1β and it inhibits translation of APP [138,139]. Posiphen is an enantiomer of phenserine and it is not an AChEI. Both posiphen and phenserine metabolites exhibit neuroprotective behaviors [140]. Furthermore, a recent study explored the relationship between phenserine and its inhibition of Pre-Programmed Cell Death (PPCD), which is a feature of Alzheimer’s and this study serves as a starting model for further research [141]. However, how phenserine promotes neuroprotection or acts as an APP translation blockade is unknown. Despite the biological benefits, there have been statistical controversies over the efficacy of posiphen and phenserine, based on their clinical trials [142]. More information is required concerning posiphen and phenserine.

Along with posiphen/phenserine, another preclinical compound mentioned previously in this review, designated as JTR-009, more effectively blocks APP translation *via* the 5’ UTR than phenserine and posiphen. JTR-009 inhibited the APP 5’UTR to directly limit Aβ [39,108]. JTR-009 has an IC_50_=100 nM inhibition value for the 5’ UTR region of APP, while posiphen has an IC_50_=5µM inhibition value [39,143]. JTR-009 is an interesting compound and more current research about its properties is required.

### Carvedilol

Carvedilol is an FDA approved agent which blocks the beta adrenergic receptor and is mainly prescribed for high blood pressure [144]. Carvedilol protects against Aluminum toxicity and a recent study has shown that aluminum oxide particles inhibit spatial learning and memory [145,146]. However, it is unclear if this drug affects iron homeostasis [145]. Carvedilol has also been shown to decrease IL-1β levels, so it could possibly promote neurogenesis because IL-1β has been shown to decrease neurogenesis [147]. Furthermore, in one study, carvedilol prevented toxicity *via* Aβ, reduced ROS levels and decreased apoptosis signaling in mice Neuro 2a (N2a) cells [148]. Carvedilol is the only screened compound of its kind for its effects on various components of AD and whether or not other beta blocker drugs such as propranolol or metoprolol could produce similar effects is unknown.

### LY450139

Pharmacologically, γ-secretase inhibitors like LY450139 (also known as Semagcestat) could potentially prevent APP progression into Aβ, previous studies have shown that γ-secretase inhibitors reduce Aβ peptides *in vitro* and one study has shown that LY450139 reduces Aβ *in vivo* [149–151]. A few studies in particular have demonstrated the positive effects of LY450139. In one study, the drug was shown to target Aβ and PS-1 by increasing the Aβ_40_/Aβ_42_ ratio [152]. Another study showed that it reduced newly synthesized Aβ peptides and yet another showed that it improved cognition in patients that had medium to high levels of Aβ [153,154].

However, there are several issues with LY450139 1) it worsened cognitive abilities in AD patients in some research settings, 2) it showed an increase in skin cancer and 3) it is neurotoxic (decreases dendritic spine density) [149,155,156]. In summary, this drug is a promising compound for the reduction of Aβ, nevertheless, like other drugs that showed initial promise, LY450139 has not been well tolerated in clinical trials, it has been terminated at phase 3 and other γ-secretase inhibitors have also been unsuccessful thus far [157–159]. Perhaps a formula that combines LY450139 with a less problematic drug would be more successful, as LY450139 does have some positive molecular capabilities but seems to be harmful to patients. One study suggests that the drug has failed in clinical trials because modulators of activity surrounding γ-secretase can aid in the progression of Alzheimer’s and that moderate inhibition of γ-secretase does not have beneficial effects, LY450139 may only be moderately inhibiting γ-secretase and this may be the reason that it has been unsuccessful [160]. Furthermore, an intriguing study complicates the discussion about this drug, as it states that Semagcestat is not actually a γ-secretase inhibitor and the results of this study warrant further investigation as to the true role of LY450139 [161]. LY450139 has some promising molecular capabilities despite its flaws, so its potential for AD should continue to be explored.

### IVIG 10%

IVIG (intravenous immunoglobulin) is an FDA approved biologic for the treatment of autoimmune diseases like Kawasaki disease [162]. A recent phase 3 clinical trial of IVIG for Alzheimer’s patients showed that the drug did not measurably improve cognition or function in a time span of 18 months, however, an older study showed that it increased Mini-Mental State Examination (MMSE) results over a 6 month period [163,164]. Moreover, many studies have indicated that IVIG treatment has many benefits for the AD brain, such as: 1) protecting against Aβ toxicity, 2) protecting against oxidative stress, 3) inhibiting levels of IL-1β and 4) promoting beneficial synaptic function [164–168]. One study has shown that IVIG treatment increased neurogenesis [10]. Studies have yet to be conducted about whether IVIG treatments may mediate or even block activation of the APP 5’ UTR specific IL-1 responsive acute box element when APP translation is induced in a pro-amyloidogenic pathway. These studies could reveal another mechanism by which immune suppressing antibodies may carry out beneficial effects for AD patients.

### Indomethacin and other NSAIDS

Neuroinflammation has been strongly implicated in AD pathology and non-steroidal anti-inflammatory drugs (NSAIDs) like Indomethacin may reduce the expression and signaling of neuroinflammatory cytokines IL-1β and IL-6 [70,169]. This reduction may help to slow the progression of AD at early stages. Indomethacin and other NSAIDs have also been shown to prevent the Aβ plaques found in AD by indirectly inhibiting the effects of Aβ aggregation *via* IL-1β and Indomethacin has been proven to reduce oxidative stress in the rat brain [170,171].

Furthermore, NSAIDS affect neurogenesis, a recent study showed that Indomethacin helped to improve decreased neurogenesis due to alcohol exposure, while another from 2017 showed that it elevated numbers of new hippocampal neurons [172,173]. A recent review discusses relevant research surrounding NSAIDS and Alzheimer’s, particularly in the context of the allegedbutsofarunclearneuroprotective role of NSAIDS and proposes that low concentrations of NSAIDS may be neuroprotective because of their inhibition of mitochondrial calcium overload [174,175]. Although indomethacin and other NSAIDS have some promising capabilities, the drugs have produced mixed effects in AD clinical trials and their precise mechanism of action on AD is still unclear, therefore, more research is required [176,177].

### Lithium carbonate (Li_2_CO_3_)

Lithium Carbonate (Li2CO3) is an FDA approved agent used to treat Bipolar disorder and other behavioral disorders. Research has shown that Lithium Carbonate can inhibit GSK-3β and GSK-3β is known to increase tau neurofibrils *via* tau phosphorylation [178,179]. In addition, an increase in GSK-3 was implicated in Aβ-induced neurodegeneration [180]. Additionally, Li_2_CO_3_ protects newly proliferated neurons and other hippocampal neurons and increases neural precursors, but it is unknown if the compound is neurogenic or if it simply increases fetal progenitors [181–183]. However, Lithium Carbonate can cause serious side effects in AD patients and a 2017 study proposed that ionic co-crystal of Lithium Salicylate and 1-Proline (LISPRO) is a more effective form of Lithium (Li) treatment for Alzheimer’s because it creates higher and more stable levels of Li, is safer for patients and significantly reduced Aβ and tau-phosphorylation [184]. More research about this alternate form of Li is needed.

Furthermore, Li is also of interest to the APP 5’ UTR translation model. It is an alkali metal that may impart anti-amyloid therapy by competing with iron to bind to the IRE RNA stem loop in APP mRNA. Therefore, it is critical to test the anti-amyloid efficacy of lithium *via* the IRP/APP IRE interaction in the 5’ UTR of APP transcript while noting that Li, at the same time, may enhance neurogenesis.

## Conclusion

AD is an extremely complex, polygenic and multifactorial disease that incorporates several biochemical proteins such as Tau, APP, Aβ, etc. Since AD is so complex, the development of new pharmaceuticals is necessary to combat such a detrimental disease. There are currently only four FDA approved medications that treat AD symptoms: donepezil, galantamine, rivastigmine and memantine. They work by either inhibiting AChE or antagonizing NMDA receptors, however, they present issues. The treatments only slow the cognitive decline by increasing ACh or attempting to prevent neural damage by Glu neurotoxicity and no current treatment can fully stop or slow down the progression of the disease. The discussed experimental treatments in this review show great promise for arresting AD pathology. However, there are also several downsides to these treatments. While many of the drugs discussed have relevant molecular properties, some have not worked well in clinical trials. In addition, there is still a lack of research about these experimental treatments. The treatments listed in this review are not the only drugs currently being tested and more research needs to be done about the effectiveness of these drugs and others in the context of AD.

Furthermore, neurogenesis in AD patients is severely reduced and most of the discussed treatments have had a positive effect on neurogenesis in some studies, however, it is not entirely known how these drugs actually affect neurogenesis. It is also unclear how these drugs affect the anatomy of the brain. Further insights into the way neurogenic processes can establish new neurons and new connections between neurons and the way that alternative drug treatments for AD affects these processes are necessary, because neurons in the brain can become so vulnerable to AD.
